# Genomic analysis of *Campylobacter fetus *subspecies: identification of candidate virulence determinants and diagnostic assay targets

**DOI:** 10.1186/1471-2180-9-86

**Published:** 2009-05-08

**Authors:** Paula M Moolhuijzen, Ala E Lew-Tabor, Bartosz M Wlodek, Fernán G Agüero, Diego J Comerci, Rodolfo A Ugalde, Daniel O Sanchez, Rudi Appels, Matthew Bellgard

**Affiliations:** 1 Centre for Comparative Genomics, School for Information Technology, Murdoch University, Murdoch, Western Australia, Australia; 2 Emerging Technologies, Queensland Department of Primary Industries & Fisheries, Yeeongpilly, Queensland, Australia; 3 Instituto de Investigaciones Biotecnológicas, Universidad Nacional de General San Martín – CONICET, 1650 KNA, San Martín, Buenos Aires, Argentina

## Abstract

**Background:**

*Campylobacter fetus *subspecies *venerealis *is the causative agent of bovine genital campylobacteriosis, asymptomatic in bulls the disease is spread to female cattle causing extensive reproductive loss. The microbiological and molecular differentiation of *C. fetus *subsp. *venerealis *from *C. fetus *subsp. *fetus *is extremely difficult. This study describes the analysis of the available *C. fetus *subsp. *venerealis *AZUL-94 strain genome (~75–80%) to identify elements exclusively found in *C. fetus *subsp. *venerealis *strains as potential diagnostic targets and the characterisation of subspecies virulence genes.

**Results:**

Eighty Kb of genomic sequence (22 contigs) was identified as unique to *C. fetus *subsp. *venerealis *AZUL-94 and consisted of type IV secretory pathway components, putative plasmid genes and hypothetical proteins. Of the 9 PCR assays developed to target *C. fetus *subsp. *venerealis *type IV secretion system genes, 4 of these were specific for *C. fetus *subsp. *venerealis *biovar *venerealis *and did not detect *C. fetus *subsp. *venerealis *biovar *intermedius*. Two assays were specific for *C. fetus *subsp. *venerealis *AZUL-94 strain, with a further single assay specific for the AZUL-94 strain and *C. fetus *subsp. *venerealis *biovar *intermedius *(and not the remaining *C. fetus *subsp. *venerealis *biovar *venerealis *strains tested). *C. fetus *subsp. *fetus *and *C. fetus *subsp. *venerealis *were found to share most common *Campylobacter *virulence factors such as SAP, chemotaxis, flagellar biosynthesis, 2-component systems and cytolethal distending toxin subunits (A, B, C). We did not however, identify in *C. fetus *the full complement of bacterial adherence candidates commonly found in other *Campylobacter *spp.

**Conclusion:**

The comparison of the available *C. fetus *subsp. *venerealis *genome sequence with the *C. fetus *subsp. *fetus *genome identified 80 kb of unique *C. fetus *subsp. *venerealis *AZUL94 sequence, with subsequent PCR confirmation demonstrating inconsistent amplification of these targets in all other *C. fetus *subsp. *venerealis *strains and biovars tested. The assays developed here highlight the complexity of targeting strain specific virulence genes for field studies for the molecular identification and epidemiology of *C. fetus*.

## Background

Northern Australian beef herds have a 35% unexplained reduction in calf production. In Argentina, calf production has not declined, but remains at a constantly low rate (63%). To aid the detection and treatment of cattle infected with *Campylobacter fetus *our genomic analysis has identified candidate subspecies specific genes that can be used as diagnostic tools.

The *Campylobacter *genus is a Gram-negative, spiral-shaped bacterium and includes 23 recorded species in the NCBI Taxonomy division. *Campylobacter *spp. colonise diverse hosts from livestock to humans with varying degrees of virulence [1]. Hosts include cattle, swine, bird, and can be the major cause of human bacterial gastroenteritis [2]. *C. fetus *subsp. *venerealis *(*Cfv*) is the causative agent of bovine genital campylobacteriosis, which causes conception failure and embryo loss, with bulls acting as asymptomatic carriers [3]. *C. fetus *subsp. *fetus *(*Cff*) causes infertility and infectious abortions in domesticated sheep, goats and cattle [2]. It is also an opportunistic pathogen in humans that can severely affect immuno-compromised patients. Initially the bacterium can cause gastroenteritis, and then spread systemically throughout the blood (bacteremia) and cause septicaemia, meningitis, and other systemic infections [2]. Bovine genital campylobacteriosis is an Office International des Epizooties (OIE) notifiable disease considered to have socio-economic and public health implications, particularly with respect to the international trade of animals and animal products [4].

Although *Campylobacter *sub species have largely conserved genomes, sub species display variable virulence phenotypes in animal models and this phenotypic virulence has been speculated to be due to hyper-variable antigenic diversity and immune evasion [1,5]. Very few gene targets have been identified for the differentiation of *C. fetus *subspecies, with members of the subspecies shown to be 86% similar based on PFGE-DNA profiles [6]. Diagnostic testing of *C. fetus *colonies from transport medium and the biochemical differentiation of the 2 subspecies *venerealis *and *fetus *is important for the diagnosis of bovine venereal disease in cattle. *Cff *and *Cfv *can be differentiated from each other using a range of biochemical assays including H_2_S, selenite reduction, growth at 42°C, susceptibility to metronidazole and cefoperazone, basic fuchsin, KMnO_4 _and glycine tolerance [6,7]. Glycine tolerance is the OIE recommended assay. It is however difficult to isolate viable colonies from transport medium for biochemical analysis due to prolonged transport, contaminant overgrowth and the fastidious nature of the bacteria [8-10]. In addition doubts in regard to the stability of these biochemical markers has been suggested based on evidence from phage transduction [6,11-13]. The H_2_S test although described as differentiating *Cff *(positive) and *Cfv *(negative), a *Cfv *strain subsequently named *Cfv *biovar *intermedius *is positive in this assay [14]. Molecular typing methods such as amplified fragment length polymorphism (AFLP) and multilocus sequence typing have been developed to differentiate *C. fetus *isolates [11,15], but these methods require the isolation of pure colonies which are impractical for diagnostic application. Specific polymerase chain reaction (PCR) assays have been designed and applied to detect *Cfv *[16-18], however it has been suggested that the gene targets are plasmid borne and that in some cases have not reliably detected all *Cfv *isolates [19]. A sensitive real time assay designed to target the *parA *gene originally targeted by the Hum et al (1997) PCR assay, identified a high prevalence of *Cfv *in Australia cattle not associated with venereal cases [4]. It was thus postulated that isolates of *Cfv *differ in virulence and that other methods may be required to confirm the presence of pathogenic *Cfv *in clinical samples. Genomic *Campylobacter *comparisons of *C. fetus *subspecies and a list of *Cfv *specific genes will provide the basis for developing specific diagnostic assays and improving our understanding of *C. fetus *virulence and epidemiology. No studies to date have reported the putative identification or extensive analysis of *Cfv *virulence genes.

Based on comparative analysis on recently available genome data for both *C. fetus *subsp. *venerealis *(*Cfv*) (incomplete) and *C. fetus *subsp. *fetus *(*Cff*) we have developed a number of assays targeting virulence factors previously identified in *C. jejuni*, *C. coli*, *C. lari*, and *C. upsaliensis *genomes. These virulence mechanisms include motility, chemotaxis, adhesion, invasion and toxin production and regulation by two-component systems, as discussed in Fouts et al [1]. This paper provides the first detailed analysis of available genome sequences in order to identify targets for differentiating *C. fetus *subspecies. Based on the analysis several targets were identified and confirmed using PCR assays.

Our aims were to (1) identify and compare *C. fetus *putative virulence genes, (2) characterise genomic features to differentiate the highly conserved *C. fetus *subspecies for diagnostic assays. The genomic features of *Campylobacter *provided subspecies markers that discriminate *C. fetus *species and subspecies, in particular the *C. fetus *sub species (*Cfv *and *Cff*) from each other and other *Campylobacter *species.

## Results

### Assembly of *Cfv *for Identifying Targets for Diagnostics

**Figure 1:**
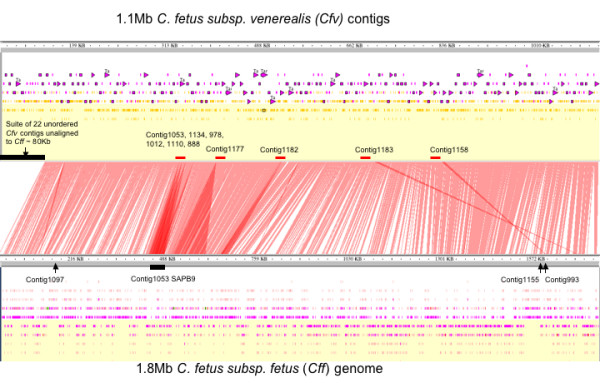
**Genomic nucleotide alignment of *C. fetus *subsp. *venerealis *(*Cfv*) contigs to the *C. fetus *subsp. *fetus *genome**. Genomic nucleotide comparison of *C. fetus *subsp. *venerealis *(*Cfv*) contigs (1.08 Mb) as aligned to the *C. fetus *subsp. *fetus *(*Cff*) completed genome (1.8 Mb). Orange shaded regions between the parallel sequences of *Cfv *(top) and *Cff *(bottom) highlight contigs in common and unique between the two *Campylobacter *subspecies.

The available genomic sequence information (ca 75–80% *Cfv *genome) was compiled using the complete *Cff *82-40 genome sequence (NC_008599) in order to identify targets for the diagnostics for detecting *Cfv*. The ordering of available genome segments generally aligned well with the *Cff *genome as shown in Figure 1.

Several striking features were evident in the subspecies comparison. Firstly, an 80 Kb suite of 22 *Cfv *specific contigs (relative to *Cff*) housed a range of putative virulence factors such as Type IV secretion systems (Additional file 1). Secondly a number of potential virulence factors were also identified in the genomic sequences that were shared between *Cfv *and *Cff *(Additional file 2). Table 1 summarises virulence factors by comparing the ORFs of the 2 *C. fetus *subspecies with 4 *Campylobacter *species as described in Fouts et al (2005). In general similar numbers of genes potentially associated with 2 component systems, toxin production, outer membrane proteins, and motility were identified. Only one bacterial adherence gene was identified in both *C. fetus *subspecies with 2 and 3 ORFs identified in *Cfv *and *Cff *respectively (Table 1). However an additional adhesion homologue fibronectin (FN) binding ORF was identified in *Cfv *but not *Cff*. A large surface array protein was found highly conserved in both species (not shown in this study) but was evident in the genomic sequence alignments (figure 1).

**Table 1:** *C. fetus *subsp. *fetus *(*Cff*) and subsp. *venerealis *(*Cfv*) virulence factors compared with 4 other *Campylobacter *spp.

Putative virulence type	**Other spp**.^a^	*Cff*	Cfv*
Bacterial adherence	9	3^b^	4^b^

Motility	55–66	41	46

Two-component system genes	11–15	16	14

Toxin and resistance	15–20	9^c^	7^c^

Membrane proteins	185–218	209	202

Summary of *C. fetus *virulence gene ORFs in *C. fetus *subsp. *fetus *(*Cff*) and subsp. *venerealis *(*Cfv*) compared with 4 other *Campylobacter *spp. (adapted from Fouts et al).

^a ^*C. jejuni, C. lari, C. upsaliensis, C. coli *(Fouts *et al*. 2005)

^b^*Cff *– PEB1 (3) – no other adherence homologues found; *Cfv *ORFs – PEB1(2), *cadF*(0), *jlpA *(1-poor homology), Fibronectin binding (1), 43-kDA MOMP (0)

^c ^not including resistance genes for *Cff *and *Cfv*, toxin subunit ORFs only

*N.B. *Cfv *genome incomplete

The nucleotide alignment of *Cfv *contigs based on the closest sequenced genome *Cff *displayed the *Cfv *contig sequence in common between the two genomes (not specific to *Cfv*) and *Cfv *contig sequence not found in *Cff *(specific to *Cfv) *(Figure 1). Of the 273 *Cfv *contigs, 251 contigs (993569 bp) were conserved with *Cff *and 22 contigs (86999 bp) specific to the *Cfv *genome compared to *Cff*. Contigs specific to *Cfv *were Contig1018, Contig1021, Contig1023, Contig1024, Contig1030, Contig1031, Contig1042, Contig1120, Contig1139, Contig1165, Contig1181, Contig1185, Contig1186, Contig419, Contig733, Contig846, Contig851, Contig872, Contig875, Contig914, Contig958 and Contig991 (ORF without strong homology to *Cff *are listed in Additional file 1).

When probed against all available genome protein sequence information the *Cfv *specific contigs (Additional file 3: Table S1) had the following alignments; two contigs (~4.9 Kb) with short alignments to only non-campylobacter bacterial species (Contigs914 and 875) (*Campylobacter *specific); five contigs (~20 Kb) with significant alignments to *C. jejuni *and *C. coli *plasmid genomes and short alignments to *C. hominis *and *C. lari*; ten contigs completely unique to *Cfv *(*Cfv *specific) (~32 Kb); and five contigs (~27 Kb) with significant protein alignments to *Cff *although this was not evident at the nucleotide sequence level.

### *Cfv *Open Reading Frame Analysis

The *C. fetus *subsp. *venerealis *1474 ORFs protein database search found 67 unique to *Cfv *(no protein alignments), 1174 conserved top match alignment to *Cff*, 116 conserved top match alignment to any other species, and 117 low significance alignments. ORF alignments to the non-redundant protein database found 12% *Cfv *insignificant and unique (Additional file 1), 51% with significant alignments and 37% with highly significant alignments. Comparison of the 9 *Campylobacter *genome protein datasets found approximately 50% of proteins were in common for all *C. jejuni *(including subsp. *jejuni *and *doyley*) except *C. jejuni *subsp. *jejuni *81–116 which had 20–25% similar. This level of similarity was also found between the *Cff *subspecies while between all *Campylobacter *species this similarity decreased to between 0.5–5.5%. The BlastMatrix [20] result can be found in additional file 4.

### *Cfv *Open Reading Frame Analysis of the *Cfv *specific suite of genomic regions

Eighteen *Cfv *specific contig ORFs were analysed against all available protein datasets. These *Cfv *specific regions contained 90 ORFs, 15 with alignments to hypothetical proteins, 32 with non-significant protein alignments and 43 ORFs with significant alignments. As a separate category these latter 43 ORFs were found to have significant alignments to plasmid/phage like proteins within *Campylobacter *species (34 ORFs) and to other bacteria (9 ORFs). In the 34 *Campylobacter *ORFs, best matches were found in two *Cfv *ORFs, namely a putative type IV secretion system protein identified in Is*Cfe*1 [18] and a putative TrbL/VirB6 plasmid conjugal transfer protein. The remaining 32 ORFs had significant hits to *Campylobacter *species other than *Cfv *such as *C. curvus *(1), *C. concisus *(2), *C. coli *(4), *C. fetus *(5), *C. jejuni *(13) and *C. hominis *(17).

Functional assignments for the *Cfv *specific ORFs were as follows; cellular processes and signalling, chromosome partitioning, cell motility and intracellular trafficking, secretion and vesicular transport (16); information storage and processing, replication, recombination and repair, transcription, translation (12); metabolism and transport amino acid, carbohydrate and inorganic ion, energy production and conversion (5); and poorly characterized, general function prediction only (7) (Additional file 1).

### *Cfv *ISC*fe1 *insertion elements

Specific sites previously identified for the IS*Cfe*1 insertion element [18] were searched in *Cfv *alignments to *Cff *(Figure 1): (a) the sodium/hydrogen exchanger protein gene *nahE *(YP_891382) was found in the *Cfv *pseudomolecule positioned 159601–160764 bp (Contig1097), a region conserved with *Cff*; (b) the putative methyltransferase protein gene metT (YP_892765) was found in the *Cfv *pseudomolecule positioned 1605092–1603530 bp (Contig1155) a region also conserved in *Cff*; and (c) the putative VirB6 protein gene was found in a number of *Cfv *contigs, these include contigs with ORFs not common with *Cff *Contig1023 and *Cfv *specific Contig1165, Contig733, Contig875 and Contig958.

*Cfv *contigs were searched for the IS*Cfe*1 insertion containing sequences (AM260752, AM286430, AM286431 and AM286432). All the IS*Cfe*1 sequences aligned to Contig993 (39–1464 bp) with greater than 90 percent identity. Contig993 *Cff *position is indicated in figure 1. The IS*Cfe*1 genes *tnpA *and *tnpB *matched Contig993 orf1 partial transposase A (*Cfv*) (14–157) and Contig993 orf2 transposase B (*Cfv*) (144–1436). Upstream ORF regions in Contig993 are: Contig993 orf3 anaerobic C4-dicarboxylate transporter (*Cff*) 1509–1697 bp; Contig993 orf4, anaerobic C4-dicarboxylate transporter (*Cff*) 1705–2493 bp; and Contig993 orf6 which had no protein alignments 2795–2968 bp.

Contig875 only aligned with AM286432 (21–1235 bp) putative virulence genes with >90% sequence identity. Contig875 orf3 (499–1068 bp) partially to the partial putative virulence gene VirB5 and Contig875 orf5 (1302–2069 bp) to the truncated putative TrbL/VirB6 plasmid conjugal transfer (*Cfv*) gene. Downstream in Contig875 were Contig875 orf1 transposase OrfA (*Helicobacter pylori*) 30–170 bp and Contig875 orf2 (274–489 bp) with no protein alignments.

### Genomic Plasmid Analysis

Plasmid containing *Campylobacters *include *C. coli*, *C. lari, C. concisus *13826 (2 plasmids), *C. hominis *ATCC BAA-381 (1 plasmid), *C. jejuni *subsp. *jejuni *81–176 (2 plasmids) and *C. fetus *subsp *venerealis *strain 4111/108. Complete plasmids have been sequenced for *C. coli *(6), *C. lari *(2), other *C. jejuni strains *(6) and *C. fetus *subsp *venerealis *(1). A direct search of these extrachromosomal *Campylobacter *plasmid sequences against *Cfv *specific sequence determined plasmid borne genes in common between the species. Plasmid sequences from *C. coli, C. hominus *and *C. jejuni *represent over a third of the *Cfv *specific ORFs (37/90). These include type IV secretion system (Vir and Cmg), ParA, Ssb, RepE, moblization and plasmid (Cpp and pTet) proteins (Additional file 3: Table S2). Tranposase genes were absent in the other *Campylobacter *spp. plasmids and found in *Cfv *Contigs1185 (2), Contig872 (1) and Contig875 (1). The *C. fetus *subsp *venerealis *plasmid *pCFV*108 (EF050075*) *contains four genes, putative *mobC*, putative *mobA*, repE and an uncharacterised orf3 [21]. Plasmid pCFV108 ws not found in the *Cfv *contigs. A protein search however found significant alignments for Contig1185.orf00004 to MobA (ABK41363 489 aa) and Contig1185.orf00007 to RepE (ABK41364 351 aa) (Additional file 5)

### COG Analysis -Virulence Genes

The String database analyses identified 1141 *Cfv *ORFs that aligned significantly to String assigned COG functions. Comparative analysis between *Cfv *to the Cluster Orthologous groups found 273 ORF in cellular processing and signalling a COG role known to contain virulence determinants, 164 information storage and processing, 406 metabolism, 153 poorly characterised, 87 to hypothetical proteins and the remaining without assignments to COG roles.

COG role distributions for virulence ORFs can be found in additional file 2.

In putative virulence roles, 49 Cfv ORFs are involved in cell motility, 83 in cell wall/membrane/envelope biogenesis, 21 defence mechanisms, 25 intracellular trafficking, secretion and vesicular transport and 29 signal transduction mechanisms.

To identify virulence genes unique to *Cfv *or other *Campylobacter *species and distinguish the two subspecies, the *Cff *and *Cfv *virulence genes and *Cfv *contigs were aligned to the *Cff *genome. The non-redundant protein search also identified virulence genes such as Cytolethal distending toxin proteins (Cdt) [1], currently characterised as a hypothetical protein within the non-supervised orthologous groupings of String, although characterized and reported by Asakura et al in 2007 [22,23].

Based on COG analyses (Additional files 1 &2) the following sequences found in categories that were both specific and not specific to *Cfv*, were selected for PCR validation. Those selected for PCR included virulence genes (including the Type IV secretion genes specific to *Cfv*) (8), flagella (6), cytolethal distending toxin (3), response regulator-sensor (6), membrane (4), fibronectin (1), haemolysin (1), Fe ABC transporter (1) and mannose-1-phosphate guanylyltransferase/mannose-6-phosphate isomerase (1) genes (Additional file 3: Table S3).

### PCR Results

**Figure 2:**
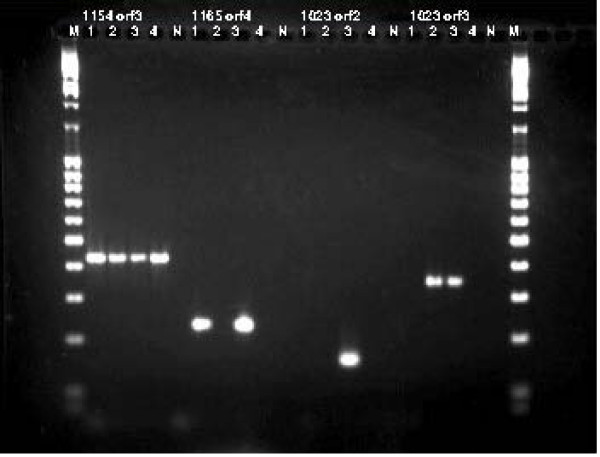
**PCR assay specificity for *C. fetus *subspecies and *C. fetus *subsp *veneralis***. Examples of PCR assay specificity for *C. fetus *subspecies and *C. fetus *subsp *veneralis *biovars (*venerealis *and *intermedius*). Lanes numbered 1–4, N and M represent: 1 *Cfv *biovar *venerealis *19438 ATCC, 2 *Cfv *biovar *intermedius *(Pfizer strain), 3 *Cfv *Argentina AZUL-94 strain, 4 *Cff *15296 ATCC, N= negative no template control and M = molecular weight marker 100 bp ladder (Invitrogen). Results are shown for assays based on Contig1154 orf3 (429 bp), Contig 1165 orf4 (233 bp), Contig 1023 orf2 (159 bp) and Contig1023 orf3 (349 bp).

To validate the subspecies specificity of virulence genes and *Cfv *specific sequences identified above, 31*Cfv *ORF sequences were selected in *Cfv *and primer sets tested using *Cff *and *Cfv *isolates (Additional file 3: Table S3). Reference and type strains screened are described in Table 2 and *Cfv *reference strains included 4 *Cfv *biovar *venerealis *isolates (DPI, ATCC, UNSAM and Pfizer) and a *Cfv *biovar *intermedius *(Pfizer) isolate. *Cff *strains used were DPI and ATCC isolates as described in Table 2. All primers were based on the *Cfv *biovar *venerealis *AZUL-94 strain contig sequences except for *flhA *and *flhB *which were based on *Cff *sequence for these 2 flagella genes not identified in *Cfv *contigs. Conserved amplification of virulence genes in both *C. fetus *subspecies included flagella, outer membrane proteins, 2 component systems (response regulators and sensors), haemolysin, iron uptake and a fibronectin type III domain protein (Additional file 3: Table S3). For assays based on ORFs selected as absent in *Cff*, contigs 1120 orf4, 1165 orfs 4, 8 and 875 orf5 assays amplified the *Cfv *biovar *venerealis *strains but not *Cfv *biovar *intermedius *or the *Cff *reference strains. These contigs were identified as: VirB4, VirB11, VirD4 and VirB6 type IV secretion system proteins respectively. Three assays (1023 orf2/VirB10, 1023 orf3/VirB11 and 733 orf1/VirB4) were specific for *Cfv *biovar *venerealis *AZUL-94 strain and did not amplify other biovar *venerealis *strains. One of these assays Contig 1023 orf 3 (VirB11) also amplified *Cfv *biovar *intermedius*. *Cfv *biovar *intermedius *was negative in all other '*Cfv*' specific assays, which in the current study appear to be specific for *Cfv *biovar *venerealis*. Curiously, 1 assay based on 1165 orf 2 (*Cfv VirB9*) was positive for *Cfv *biovar *venerealis *AZUL-94, *Cfv *biovar *intermedius *and both *Cff *strains tested but did not amplify the other 3 *Cfv *biovar *venerealis *strains including the ATCC 19438 strain. All assays were specific for *C. fetus *subspecies, testing negative in related strains and reproductive disease pathogens listed in Table 2 including: *C. coli*, *C. jejuni, C. sputorum subsp. bubulus, C. hyointestinalis, Pseudomonas aeruginosa, Proteus vulgaris, Neospora caninum and Tritrichomonas foetus *(results not presented). However no single assay amplified all *Cfv *strains inclusive of both biovars *venerealis *and *intermedius*. Figure 2 demonstrates the specificity of selected primer sets Contig1023 orf2 and orf3, Contig1154 orf3 and Contig1165 orf4. Contig1023 orf3 and Contig1165 orf4 primers amplified sequences specific for *Cfv*, while Contig1154 orf3 primers amplified sequences in both *Cfv *and *Cff *strains.

**Table 2:** Reference strains tested in *C. fetus *PCR assays

Species and subspecies	Strain	Source^1^
*C. fetus *subsp. *venerealis*	98–109383 (Biovar *venerealis*)	Field Isolate (DPI&F, QLD)
*C. fetus *subsp. *venerealis*	19438 (Biovar *venerealis*)	ATCC 19438
*C. fetus *subsp. *venerealis*	AZUL-94 (Biovar *venerealis*)	UNSAM, Argentina
*C. fetus *subsp. *venerealis*	Biovar *venerealis*	Pfizer Animal Health
*C. fetus *subsp. *venerealis*	Biovar *intermedius*	Pfizer Animal Health
*C. fetus *subsp. *fetus*	98–118432	Field Isolate (DPI&F, QLD)
*C. fetus *subsp. *fetus*	15296	ATCC 15296
*C. coli*	11353	NTCC
*C. jejuni *subsp. *jejuni*	11168	NTCC
*C. hyointestinalis*	N3145	Field Isolate (DPI&F, QLD)
*C. sputorum *subsp. *bubulus*	Y4291-1	Field Isolate (DPI&F, QLD)
*Pseudomonas aeruginosa*	27853	ATCC
*Proteus vulgaris*	6380	ATCC
*Neospora caninum*	50843	ATCC
*Tritrichomonas foetus*	YVL-W	Field Isolate (DPI&F, QLD)

^1^Legend: ATCC – American Type Culture Collection; NTCC – National Type Culture Collection; UNSAM – Universidad Nacional de General San Martín; DPI&F – Department of Primary Industries and Fisheries

## Discussion

The available *Cfv *genomic sequence information was aligned to the complete *Cff *genome sequence 82–40 in order to identify targets for the diagnostics for detecting *Cfv*. Based on the genome size estimates of *Cfv *[6,24] and the completed *Cff *genome size, it is estimated that approximately 72% of the *Cfv *genome has been sequenced (unpublished, Prof Daniel Sanchez, Universidad Nacional de San Martin, Argentina). The ordering of available genome segments generally aligned well with the *Cff *genome as shown in Figure 1 and made evident a suite of *Cfv *specific contigs. This suite of contigs housed a large range of type IV secretion factors, and plasmid/phage like proteins. A number of potential virulence factors were clearly identified as shared between *Cfv *and *Cff*. These virulence factors include an outer surface array membrane protein, chemotaxis types, motility associated, regulatory and secretion systems. The existence of these classes of genes with roles in the infection process, but not showing sub species specificity, is consistent with a two-tier infection model. Surface/membrane components provide necessary (but not sufficient) structural components for attachment to host cells. Specific components that complete the features of the surface/membrane structures are required for infection. Fouts et al., (2005) found that many genes involved in host colonization were conserved across the *Campylobacter *genus. Variations that were species specific were evident for a lipo-oligosaccharide locus, a capsular (extracellular) polysaccharide locus, and a novel *Campylobacter *putative licABCD virulence locus (not found in available *Cfv*). These observations are consistent with the suggestions that interactions between a pathogen's surface-exposed proteins and host cells represent a pivotal step in pathogenesis and virulence [25]. In pathogens several of the key players are proteins involved in adhesion, invasion, secretion, signalling, annulling host responses, toxicity, motility and lipoproteins [26].

Motility and chemotaxis genes have been found conserved among related *Campylobacter *species with flagella implicated in adhesion, protein secretion, invasion and virulence in pathogenic *C. jejuni *[1,27-30]. Biosynthesis of flagella requires the involvement of more than 40 structural and regulatory proteins including a type III secretion system for flagellar assembly [28,30-32]. The *Cff flhA *gene based on genome alignments was found to be absent in the available *Cfv *sequence contigs, and coincided with the ordered alignment gap/non-sequenced section relative to *Cff*. However, one chemotaxis regulatory protein campy.fasta.screen.Contig1091 orf6 appears to be absent in *Cff *(Additional file 1). We identified a lower complement of homologues associated with motility in *Cff *(n = 41) compared with the other *Campylobacter *spp. (n = 55–66) [1], however, the analysis of the incomplete *Cfv *genome identified a higher number of homologues (n = 46) than the total *Cff *sequence. PCR assays based on a subset of flagellar genes (*flgH*, *flhF*, *fliH*, *flhA *and *fhlB*), demonstrated conservation of these sequences at least among the members of our panel of *C. fetus *strains including both subspecies (although *flhA *could not be identified in the available *Cfv *contigs). An additional assay designed to amplify the *flaB *sequence of the *Cfv *AZUL-94 strain did not amplify other *Cfv *biovar *venerealis *strains but did amplify *Cfv intermedius *and the *Cff *isolates. We have not confirmed if this is attributed to *flaB *sequence variation or an absence of the gene in different geographical *Cfv *biovar *venerealis *strains, this gene has been targeted however for genotyping studies in other *Campylobacte*r species [33]. This study does confirm that the complete *Cfv *genome may harbour more flagellar/motility homologues than *Cff*. Virulent *C. jejuni *harbours more flagellar genes than less virulent species *C. coli*, *C. lari *and *C. upsaliensis *[1].

Adherence of other *Campylobacter *species to gut epithelial cells is mediated by multiple adhesins including *cadF *(*Campylobacter *adhesion to fibronectin); [34], PEB1 protein (putative binding component of an ABC transporter), [35], *JlpA *(jejuni lipoprotein A), [36] and a 43-kDa major outer membrane protein [37], confirmed as conserved in *C. jejuni*, *C. lari*, *C. upsaliensis *and *C. coli *genomes [1]. *Cfv *homologues for PEB1 and fibronectin-binding (FN-binding) proteins were confirmed with the remaining 3 absent in the genome contigs currently available. However, only the PEB1 protein was identified in the complete *Cff *genome sequence 82–40. Fibronectin is known to enhance *C. fetus *attachment [38] however in the absence of an identified *C. fetus cadF *homologue, it appears that the adherence mechanisms in *C. fetus *may differ from other *Campylobacter *species. In the case of *C. fetus *subsp. *venerealis*, this is perhaps not surprising as *Cfv *colonise the genital tract and not the intestinal tract, thus perhaps novel adhesins will be identified with completion of a *Cfv *genome sequence.

Toxin sequences, two component regulatory systems, plasmids and type IV secretion systems have also been recognised as components in pathogenic *Campylobacter *spp. [1]. Three cytolethal distending toxin (*cdt*) subunits A, B and C are confirmed as conserved across the four *Campylobacter *species (*C. jejuni, C.lari, C. coli, C. upsaliensis*) and *C. fetus *[22,23]. In addition, the presence of *cdt *genes is linked to *C. jejuni, C coli *and *C. fetus *pathogenesis, where *cdt *negative strains were found to be less efficient during adherence and invasion *in vitro *[22,39]. A similar survey of *C. fetus *will assist to confirm if *cdt *positivity is associated with an increase in pathogenicity. Two-component regulatory (TCR) systems are commonly used by bacteria to respond to specific environmental signals such as temperature [40]. Five TCR systems (pairs of adjacent histidine kinase and response regulator genes) have been identified as conserved across *Campylobacter *species and confirmed in *C. fetus *subspecies.

The type IV secretory genes, which are possibly involved in conjugative plasmid transfer or the secretion of virulence factors [1,18,41], were absent in the *Cff *genome and unique to *Cfv*. A large proportion of *Cfv *subspecies specific ORFs (30%) were harboured in the *Cfv *contig specific regions. *C. upsaliensis *and *C. jejuni *are known to harbour plasmids and evidence does suggest that these plasmids can play a role in pathogenesis. One basic difference between the list of genes absent in *Cff *and present in *Cfv *is that many of them are in common to genes present on the plasmids of these related *Campylobacter*. The type IV secretion system is also found in *C. jejuni*, *C. lari *and *C. coli *plasmid sequence. The unique *Cfv *genome sequences also harboured many phage-like derived genes. The presence of type IV secretion system has also been described by Abril et al, 2007 [18], of which the putative VirB6 protein gene was found to be truncated by the insertion element (IS*cfe*1). It is possible that contigs within this *Cfv *unique 80 Kb suite of contigs represent a number of extrachromosomal DNA plasmids. A wider survey of *C. fetus *isolates and the presence of plasmids (type IV secretion systems) and phage genes will assist to confirm our observations.

This analysis has provided diagnostic markers to discriminate the *Campylobacter *subspecies *Cfv *and *Cff*, which can be investigated for more general applicability for field use. Most of the *Cfv *assays based on the incomplete AZUL-94 genome sequence, showed amplification preference for *Cfv *biovar *venerealis *strains. The *Cfv *biovar *intermedius *strains were negative in all but one assay, which was otherwise positive for *Cfv *AZUL-94 strain only. Curiously, one of the assays designed to *Cfv *AZUL-94 strain *virB9 *(type IV Secretion gene) did not amplify other *Cfv *biovar *venerealis *isolates but did amplify biovar *intermedius *and the *Cff *strains tested here. However, as described above the *Cff *genome sequence (Strain 82–40) does not appear to have type IV secretion genes. A confounding factor in interpreting this data is that different *Cff *strains may also possess putative plasmid-borne genes and these may potentially be shared between subspecies and *Cfv *biovars. The *Cfv *AZUL-94 strain could also either consist of a mix of the 2 biovars or represent a novel strain of *Cfv*. However, assays based on putative plasmid-borne genes have previously demonstrated inconsistencies when applied for subspecies identification in some regions [19]. The *parA *(plasmid partitioning protein gene), [42] assay target is thought to be plasmid borne, however evidence for plasmids containing *parA *in *Cfv *has not been confirmed to date [19,42]. Very little research has been undertaken to compare the *Cfv *biovars and the diagnostic targets reported here now need to be further tested in multiple field strains to assess the stability of these markers and therefore the genomic regions in *Cfv*. However, the results presented do suggest that the *Cfv *research community could benefit from the generation of full genome sequence from both biovars as well as isolates from different geographical continents. Our results also demonstrated putative plasmid sequences are present in *Cfv*, absent in *Cff*, suggesting plasmid profiling and sequencing from *C. fetus *subspecies, biovars and strains will assist to confirm our findings.

## Conclusion

Our assays have highlighted the complexity of virulence factor specificity within *C. fetus *subspecies and strains probably due to plasmid borne gene elements. We found that most genes important for interactions between a pathogen's surface-exposed proteins and host cells that represent a pivotal step in pathogenesis and virulence were conserved in *C. fetus*. These genes although important, did not differentiate the subspecies and therefore not the virulence factors that determined specificity. Instead we found the suite of extrachromosomal type IV secretion system (T4SS) *vir *genes specific to the *Campylobacter fetus *subspecies *venerealis *biovar *venerealis *AZUL-94 were able to consistently discriminate the *C. fetus *subspecies *fetus *in our PCR assays. Complete genomic and plasmid data will ultimately assist to develop definitive tools for comprehensive *Campylobacter fetus *subspecies differentiation.

## Methods

### Bacterial Strains, culture conditions and DNA preparation

*Campylobacter fetus subsp. venerealis *AZUL-94, an Argentinean field strain isolated from a bovine aborted fetus in 1994 was grown routinely on Tryptic Soy Agar plates or in Brain Heart Infusion (BHI) and cultivated under microaerobic conditions in anaerobic jars with CampyGen envelopes (OXOID) at 37°C. Total DNA from *Campylobacter fetus venerealis *was isolated by the classical SDS/proteinase K/Phenol/Chloroform extraction method [43]. The Pfizer stains were originally isolated by CSIRO Australia [44].

### Library construction, DNA sequencing and assembly

Genomic DNA was randomly sheared by nebulization, treated with Bal31 nuclease and blunt ended with T4 DNA polymerase. Fragments were size fractionated by agarose gel electrophoresis and ligated to dephosphorylated *Hinc*II-digested pBS plasmid. Three libraries with insert size of approximately 2 Kbp (Cf1), 4 Kbp (Cf2), and 6 Kbp (Cf3) were generated. Template preparation and DNA sequencing were performed as described [45] from randomly selected clones. Single-pass sequencing was performed on each template using T7 or T3 primer. Sequencing reads, obtained from the three genomic libraries (Cf1, Cf2, Cf3) were masked against plasmid vector and basecalled with phred (-trim_qual). Those sequences with at least 50 good quality bases after trimming were retained for assembly. After reaching ~4.5× shotgun coverage, assembly was done using the phredPhrap script provided with phrap. The autofinish functionality of consed was used to select candidate clones for re-sequencing to increase sequence coverage, decrease the number of contigs and increase the consensus quality in a number of cases. Additional information on *Campylobacter fetus venerealis *sequencing can be found in additional file 6.

### Nucleotide sequence accession numbers

Sequence data have been deposited in the WGS division of GenBank under the following accession numbers: ACLG01000001... ACLG0101187

### Genomic Data

A subset of 273 *Cfv *contig sequences (lengths greater than 2 Kb) from 1,187 the assembled contigs (Genbank ref nos) was generously supplied by the UNSAM, Argentina for this analysis. The assembled contigs have been submitted to GenBank as a part of the WGS division (GenBank: ACLG00000000 and RefSeq: NZ_ACLG00000000). All manuscript referenced contig ORFs are listed in the Additional files 1 and 2.

Completed *Campylobacter *genomic sequences were obtained from NCBI RefSeq Genome http://www.ncbi.nlm.nih.gov. All genomic detail for *Campylobacter *species listed was downloaded from NCBI http://www.ncbi.nlm.nih.gov/Genomes/ genome division, 28^th ^April, 2008. *Campylobacter *species included *C concisus *13826, *C. curvus *525.92, *C. fetus *subsp. *fetus *82–40, *C. hominis *ATCC BAA-381, *C. jejuni *RM1221, *C. jejuni *subsp. *doylei *269.97, *C. jejuni *subsp. *jejuni *81–176 and *C. jejuni *subsp. *jejuni *81116.

Alignment of *Campylobacter *genomes was conducted using BLAT [46] 90 percent identity. The BLAT results were then filtered for a minium 50% alignment. The two *C. fetus *subspecies were then displayed in Argo [47] (Figure 1).

### Alignment of genomic *Cfv *Contigs based on *Cff*

The 273 *Cfv *AZUL-94 contigs were aligned to the *Cff 82–40 *genome (NC_008599) using BLAT [46] (>90% identity). *Cfv *contigs were ordered and assembled based on the best BLAT alignments between *Cfv *and *Cff *based on *Cff *position and strand orientations into a contiguous pseudomolecule. Unaligned contigs were concatenated to the pseudomolecule linear sequence.

### *Cfv *Open Reading Frame Identification & Annotation

ORF prediction was conducted on the 273 *Cfv *using Glimmer3 [48] for ORF lengths greater than 100 nucleotide bases resulting in 1474 open reading frames (ORF). The 273 *Cfv *and 1474 ORF were subsequently screened against public NCBI protein (nr, patent), String [49], COG [50], and NCBI Conserved Domain databases with the BLAST program [40]. These results were then categorised using BIOPERL [51] scripts based on alignment percent identity (PID) and query coverage to provide the following six alignment categories, (1) known protein > 80% PID and > 80% query coverage, (2) known protein > 30% PID and > 80% query coverage, (3) hypothetical protein > 80% PID and > 80% query coverage (4) hypothetical protein > 30% PID and > 80% query coverage, (5) alignments with an expected value less than 1e-05, < 30% PID and < 80% query coverage, and (6) alignments greater than 1e-05 < 30% PID, < 80% query coverage.

### *Campylobacter *protein similarity to *Cfv *ORF

*Campylobacter *complete proteome sequence and protein detail were downloaded from NCBI http://www.ncbi.nlm.nih.gov/genomes/lproks.cgi. The 8 complete campylobacter proteome sets were compared to our *Cfv *ORF set using BlastMatrix [20] at an ARL 0.75 and an e-value < 1e-05 (results in additional file 4).

### Putative Virulence Genes

The functional categories for *Cfv *ORFs were determined based on the String Database [49] categories developed on NCBI COG database role descriptions. The main categories being Cellular processes and signaling, Information storage and processing, Metabolism, Poorly characterized, No mapping, Non Orthologous Group (NOG) and KOG (euKaryote Orthologous Group). The ORFs identified in *Cfv *were screened against the String database and alignment results were filtered using Bioperl for greater than 80% query coverage and 30% PID or with an expected value <1e-05. These ORFs were then screened against NCBI protein database to determine selected putative virulence gene representation in the *Campylobacter *genus.

### Primer Design

Primer sets were designed on *Cfv *putative virulence genes and genes unique to *Cfv *using Primer3 [52] (Additional file 3: Table S3). Primers were screened against the Cfv AZUL-94 strain and *Cff *(strain 82–40) genome data and public databases to confirm specificity. Assays were conducted in 20 μl reaction volumes, using 10 nM of each forward and reverse primer (Additional file 3: Table S3), 1 × PCR reaction buffer with 25 mM Mg^2+ ^(HotMaster *Taq *buffer, Eppendorf, Germany), 200 μM dNTPs, 1 U Hotmaster™ *Taq *DNA polymerase and 1 ng of *C. fetus *DNA. The reactions were cycled in a Gradient Palm Cycler (Corbett Research, Australia), using the following temperature profile: an initial denaturation at 94°C for 2 min, followed by 35 cycles of denaturation at 94°C for 20s, annealing at 45 to 57°C (dependent on primer pair, Additional file 3: Table S3) for 10 s, and extension at 72°C for 30s including a final single extension for 7 min at the end of the profile. Amplification products were separated in 2% TBE (89 mM Tris borate, 2 mM EDTA, pH 8) agarose gels using 100 bp ladder (Invitrogen) and were visualised under UV illumination by ethidium bromide staining. DNA preparations from strains were screened in all assays (Table 2).

## Authors' contributions

PM conducted the bioinformatics analysis and the drafting of the manuscript. AL made substantial contributions to the manuscripts conception and design, the acquisition, analysis and interpretation of the data and the drafting of the manuscript. WB carried out the molecular analysis. DS, FA, DC and RU were responsible for the sequencing and assembly of *Cfv *and provided final approval of the manuscript version to be published. RA and MB made substantial contribution to data interpretation, drafting the manuscript and its critical revision.

## Supplementary Material

Additional File 1 **List of *C. fetus *subsp. *venerealis *specific ORF and ORF protein analyses record**. The data provided represent the Blast analysis of *C. fetus *subsp. *venerealis *specific ORF against protein dataset. Table lists contig ORF, ORF contig position, protein accession, protein description, expected value of orf alignment to the protein sequence and percentage identities in the alignment. Click here for file

Additional File 2 **List of *C. fetus *virulence gene contigs targeted in PCR assays**. The data provided represent the Blast analysis of *C. fetus *subsp. *venerealis *specific ORF against protein dataset. Table lists contig ORF, ORF contig position, protein accession, protein description, expected value of orf alignment to the protein sequence and percentage identities in the alignment. Click here for file

Additional File 3 **Supplemental Tables**. Table S1, Table S2 and Table S3. Click here for file

Additional File 4 ***Campylobacter *proteome matrix analysis**. An alignment Matrix displays protein similarity between the available *Campylobacter *complete proteomes (protein) and *Cfv *ORF (translated to amino acid). Percentage gene duplication is displayed as a percentage and as a heat map within species and across species and stains. Click here for file

Additional File 5 **Plasmid pCFV108 protein alignment to *Campylobacter fetus venerealis *ORFs**. Diagram shows Plasmid pCFV108 and AZUL-94 Contig1185 ORF homology, *Campylobacter *homology is shaded in pink. Contig1185.orf00004 aligns to MobA (ABK41363) and Contig1185.orf00007 aligns to RepE (ABK41364). Click here for file

Additional File 6 ***Campylobacter fetus venerealis *genome sequencing and assembly data**. *Campylobacter fetus venerealis *genome sequencing and assembly information. Click here for file
