# Supplementary material for: Genomic analysis of Campylobacter fetus subspecies: identification of candidate virulence determinants and diagnostic assay targets
Source: BMC Microbiol. 2009 May 8;9:86. doi: 10.1186/1471-2180-9-86 (PMC2685401; doi:10.1186/1471-2180-9-86)
Supplement: Additional File 3 — Supplemental Tables. Table S1, Table S2 and Table S3. [file 1471-2180-9-86-S3.doc]

### Supplemental tables

### Table S1. *C. fetus* subsp. *venerealis* specific regions aligned to protein

Comparison of *C. fetus* subsp. *venerealis* specific sequence regions to all available genomic protein sequences.

### Table S2. *Campylobacter* plasmid gene comparison

###

Comparison of all available *Campylobacter* plasmid gene content to the *Cfv* ORFs in the 80Kb *Cfv* specific suite of contigs. Plasmid-like genes are found in common between the different *Campylobacter* plasmid genes and *Cfv* specific ORFs.

### Table S3. *C. fetus* validated primer set sequences and amplification results using *C. fetus* reference panel.

| ***Cfv* Contig gene ID** | **gene ID**  **(assay specificity^1^)** | **Primer label** | **Primer sequence 5’ to 3’** | **Anneal ^o^C** | **Amplicon (bp)** | ***Cfv* reference strains** | | | | | ***Cff*** | |
| --- | --- | --- | --- | --- | --- | --- | --- | --- | --- | --- | --- | --- |
|  |  |  |  |  |  | 19438 **biovar ven** | 98-109383  **biovar ven** | AZUL-94 **biovar ven** | **Pfizer biovar ven** | **Pfizer biovar interm** | 15296 | 98- 118432 |
| 733 orf 1 | *virB4* (*Cfv* AZUL-94 strain specific) | C733G3F C733G3R | TGATAAATAAAGAACCTGTTT TTTTTGCATAATCATTGTT | 45 | 516 | - | - | + | - | - | - | - |
| 875 orf 5 | *virB6* (*Cfv* biovar *venerealis* specific) | n875g2F n875g2R | GTGAATACACTTCAATCCG CAGTTTCCAGCATTCATAG | 49 | 185 | + | + | + | + | - | - | - |
| 878 orf 2 | *Flagellar flgH* | C878g1F C878g1R | AGAAAACGGCATAGGCGTAA AGTGCCGCTTCCGCTATAAT | 55 | 301 | + | + | + | + | + | + | + |
| 927 orf 1 | *Cytolethal distending toxin cdt*A | NC927g1F N927g1R | TCGCACGTAGTATGAACAAC CGTATAATCCTGTTCCGGTA | 45 | 268 | + | + | + | + | + | + | + |
| 927 orf 2 | *Cytolethal distending toxin cdt*C | NC927g2F NC927g2R | GCAACAGCTTCTATCTGAACAG ATCCTTTTGGAACCGTGC | 45 | 175 | + | + | + | + | + | + | + |
| 927 orf 3 | *Cytolethal distending toxin cdt*B | NC927g3F NC927g3R | GAGCGTTTGGAGCGATAA GCGGCCATAGTAGAAAATGT | 50 | 154 | + | + | + | + | + | + | + |
| 988 orf 1 | *Outer membrane protein (onpA)* | 988G1F 988G1R | TTGAAGGAAACTGTGACGAGT CATACAGGTTTGCTCTCGC | 50 | 150 | + | + | + | + | + | + | + |
| 992 orf 7 | *Fibronectin Fn3* | 992G5F 992G5R | GTTTTGGACTTTCTAATCCG AACGACATCTGTTCAGTATGAT | 50 | 100 | + | + | + | + | + | + | + |
| 995 orf 5 | Response regulator | nC995g4F nC995g4R | TCTTGATAAACTGCATAGCGCC CGCTGCTAAATGGACTTGAGAT | 47 | 138 | + | + | + | + | + | + | + |
| 995 orf 6 | Sensor | C995g5F C995g5R | TCCTTAGCTCAAATATAGTAGGATT AGGAAGTGGAATAGGTTTGTAT | 47 | 107 | + | + | + | + | + | + | + |
| 1006 orf 4 | Membrane protein | C1006G4F C1006G4R | AAGTATGGCAAACGGCG GTACGCTAATCTGTCGACTCTC | 50 | 220 | + | + | + | + | + | + | + |
| 1013 orf 3 | *Flagellar flhF* | nC1013g1F nC1013g1R | GCTTTCTAAACTTTCGCTTC ATATGCCCGCTTCTATGA | 47 | 101 | + | + | + | + | + | + | + |
| 1023 orf 2 | *virB10*  (*Cfv* AZUL-94 strain specific | nC1023g1F nC1023g1R | AGTGGTGGATTTAAAAGCGGAC GTGGTAATCAACCCATCCTTCT | 54 | 159 | - | - | + | - | - | - | - |
| 1023.orf3 (NESTED) | *virB11* (*Cfv* AZUL-94 and *Cfv* biovar *intermediu*s specific) | C1023G3F C1023G3R | ATATCAATGGAGTCTGGCAC AATGTTGTCTTACCACTGCC | 55 | 349 | - | - | + | - | + | - | - |
| 1023 orf 3 | *virB11* (*Cfv* AZUL-94 and *Cfv* biovar *intermediu*s specific) | Nc1023g4F  NC1023G4R | ACGCTGGTAGCGTAAAGCA CAACAACCTGTCTTTTGGCTC | 55 | 161 | - | - | + | - | + | - | - |
| 1034 orf 10 | Sensor | nC1034g7F nC1034g7R | GCCCATACCGAATTTTTCT ACGCCGATATATTTTTACTGG | 45 | 101 | + | + | + | + | + | + | + |
| 1034 orf 12 | Response regulator (*OmpR*) | C1034g9F C1034g9R | TTTGGGTATTTTGGATCATC AGCGGATCTAAAAGATAGAAGT | 45 | 81 | + | + | + | + | + | + | + |
| 1037 orf 1 | haemolysin secretion/activation protein, ShlB/FhaC/HecB family | C1037g1F C1037g1R | GCGATGAATATACCGTTAGAGG GAATAGTCTCGCTCGGCAT | 50 | 291 | + | + | + | + | + | + | + |
| 1040 orf 1 | Sensor histidine kinase | C1040g1F C1040g1R | ATAAGCCTACTAATCCCATCA AATGCTGCTTTTACCCAT | 48 | 132 | + | + | + | + | + | + | + |
| 1047 orf 2 | sensor histidine kinase | nC1047g1F nC1047g1R | AGGCGAGATCTTGGATCT GCGTGAGGACTTTGTGTTC | 45 | 207 | + | + | + | + | + | + | + |
| 1083 orf 2 | sensor histidine kinase | nC1083g1F nC1083g1R | TACAAATCACAAGACTACG CGAACTTACTATGAGT | 50 | 102 | + | + | + | + | + | + | + |
| 1095 orf 4 | iron uptake ABC transport | NC1095g2F NC1095g2R | AAAGTCTCTCATCAGTCG TACGCTCTTGATAGTGGT | 55 | 250 | + | + | + | + | + | + | + |
| 1120 orf 4 | *virB4* (*Cfv* biovar *venerealis* specific) | C1120G2F C1120G2R | TTCTCCTGCAACTGACGC GCTTTAACACGTCCGCC | 50 | 521 | + | + | + | + | - | - | - |
| 1143 orf 3 | *omp* | C1143G5F C1143G5R | GGCTTTAGAGGTACGGCTCC TAACGGACGTATCATACGCG | 57 | 150 | + | + | + | + | + | + | + |
| 1154.orf3 | mannose-1-phosphate guanylyltransferase/mannose-6-phosphate isomerase | C1154g3F  C1154g3R | AAAAGCTGCAGTAGAGTTGG TCATCGAAACTTCCCATATC | 55 | 429 | + | + | + | + | + | + | + |
| 1155 orf 4 | *flaB* (*Cfv* AZUL-94, *Cfv* biovar *intermedius*, and *Cff* specific) | C1155g3F C1155g3R | ACTACCGCTTTGAGCAAGGA GGCGGTGCTAACGTATCATT | 45 | 492 | - | - | + | - | + | + | + |
| 1165 orf 2 | *virB9* (*Cfv* AZUL-94, *Cfv* biovar *intermedius*, and *Cff* specific) | nC1165g2F nC1165g2R | TGACAAAGATGAGCGGATAG TACCTGTTCGCCGTTTTC | 50 | 151 | - | - | + | - | + | + | + |
| 1165 orf4 | *virB11* (*Cfv* biovar *venerealis* specific) | nC1165g4F  nC1165g4R | AGGACACAAATGGTAACTGG GATTGTATAGCGGACTTTGC | 57 | 233 | + | + | + | + | - | - | - |
| 1165 orf 8 | *virD4*(*Cfv* biovar *venerealis* specific) | nC1165g6R nC1165g6R | ATGTTCTAGCAGAGCTTGG TGACATTACGCCACTCTT | 50 | 101 | + | + | + | + | - | - | - |
| 1172 orf 10 | *Flagellar fliH* | nC1172g7F nC1172g7R | GCTTAAAACTATAACTCCGCCG TGCTAAAAGCTTGATCAGCG | 47 | 160 | + | + | + | + | + | + | + |
| *C. fetus* subsp. *fetus* | *Flagellar flhA* | nCFFFlhAF nCFFFlhAR | TTAAGCGAAGGCCATAATGG GTTTTCCAGGCATAGCATCA | 50 | 202 | + | + | + | + | + | + | + |
| *C. fetus* subsp. *fetus* | *Flagella flhB* | C999F C999R | CTGCGGTAGGGATATTTTGC TCCACTCAATGCTTCAGACG | 45 | 543 | + | + | + | + | + | + | + |

^1^Assay specificity descriptions when not all reference strains are positive
