# Supplementary figures and images for: Genomic analysis of Campylobacter fetus subspecies: identification of candidate virulence determinants and diagnostic assay targets
Source: BMC Microbiol. 2009 May 8;9:86. doi: 10.1186/1471-2180-9-86 (PMC2685401; doi:10.1186/1471-2180-9-86)

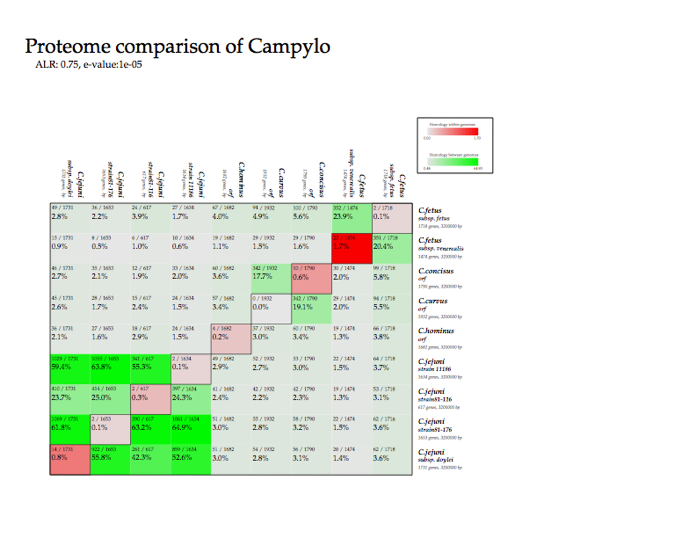

Supplement: Additional File 4 — Campylobacter proteome matrix analysis. An alignment Matrix displays protein similarity between the available Campylobacter complete proteomes (protein) and Cfv ORF (translated to amino acid). Percentage gene duplication is displayed as a percentage and as a heat map within species and across species and stains. [file 1471-2180-9-86-S4.png]

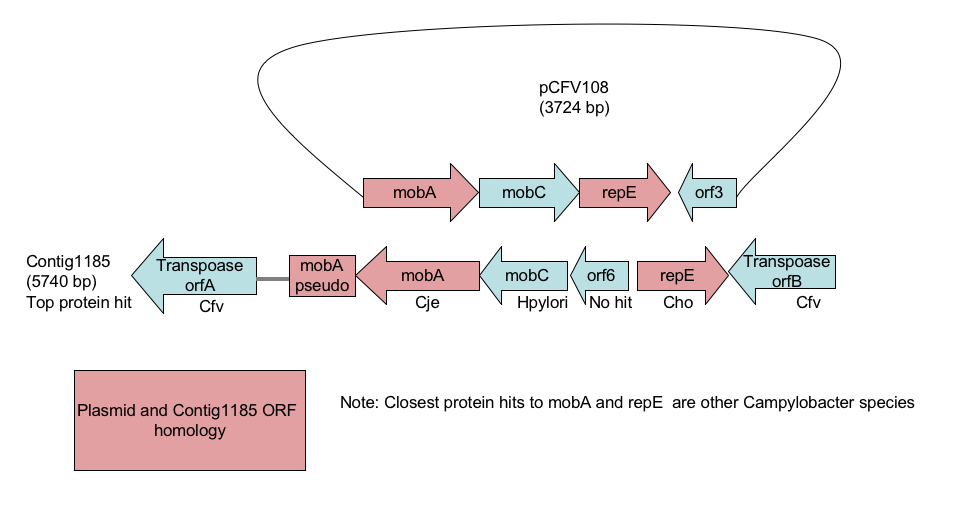

Supplement: Additional File 5 — Plasmid pCFV108 protein alignment to Campylobacter fetus venerealis ORFs. Diagram shows Plasmid pCFV108 and AZUL-94 Contig1185 ORF homology, Campylobacter homology is shaded in pink. Contig1185.orf00004 aligns to MobA (ABK41363) and Contig1185.orf00007 aligns to RepE (ABK41364). [file 1471-2180-9-86-S5.png]
