# Supplementary material for: Genomic analysis of Campylobacter fetus subspecies: identification of candidate virulence determinants and diagnostic assay targets
Source: BMC Microbiol. 2009 May 8;9:86. doi: 10.1186/1471-2180-9-86 (PMC2685401; doi:10.1186/1471-2180-9-86)
Supplement: Additional File 6 — Campylobacter fetus venerealis genome sequencing and assembly data. Campylobacter fetus venerealis genome sequencing and assembly information. [file 1471-2180-9-86-S6.doc]

**Supplementary data (S4)**

***Campylobacter fetus venerealis* sequencing.**

**Whole genome shotgun**

**Clone size estimate:** 1,342,601 bp

**Coverage:** 4.7 X

**Genomic libraries.**

**Cf1:** average insert size of 2 Kb (max 4 Kb)

**Cf3:** average insert size of 4 Kb (max 6 Kb)

**Cf2:** average insert size of 6 Kb (max 8 Kb)

**Sequencing.**

**Chemistry:** Big-Dye

**Number of reads:** 13,671

**Average read length:** 867 bp

**Reads by library:** 6748 (cf1), 4495 (cf2), 2428 (cf3)

**Reads by direction:** 7001 forward (51%), 6670 reverse (49%)

**Overall base composition:** 29.9% A, 18.3% C, 19.5% G, 30.3% T

**Assembly.**

**Contigs:** 1187

**Contigs > 2 Kb:** 273

**Singlets:** 1335
